# Determination of Mycotoxin Contamination Levels in Rice and Dietary Exposure Assessment

**DOI:** 10.1155/2022/3596768

**Published:** 2022-09-02

**Authors:** Jose Troestch, Stephany Reyes, Aracelly Vega

**Affiliations:** Centro de Investigación en Recursos Naturales, Universidad Autónoma de Chiriquí, David 0427, Chiriquí, Panama

## Abstract

The contamination by aflatoxins, ochratoxin A, and zearalenone of samples of paddy and polished rice stored in silos located in Chiriquí, Panama, was evaluated. A total of 23 samples were extracted using immunoaffinity columns and analyzed by high-performance liquid chromatography (HPLC) with a fluorescence detector (FLD) and post-column photochemical derivatization. For the method used, the detection limits were lower than 0.25 *μ*g/Kg for aflatoxins (AFB_1_, AFB_2_, AFG_1_, AFG_2_) and ochratoxin A and 9.35 *μ*g/Kg for zearalenone; the limits of quantification were between 0.25 and 18.75 *μ*g/Kg, respectively. Of the samples analyzed, all of the paddy rice samples were positive for at least one of the mycotoxins studied, zearalenone being the one found with the highest incidence (90.91%); for the polished rice samples, the mycotoxin with the highest incidence was zearalenone (50%), although in concentrations lower than those established in European legislation (100 *μ*g/Kg). The estimate of the daily zearalenone intake according to the concentrations found was always less than 0.07 *μ*g/Kg/bw. This is the first report on the determination of 6 mycotoxins in rice grains from Panama by the HPLC-FLD methodology. Considering the high incidence of mycotoxins in the analyzed rice samples, regular control in the production process is recommended to improve quality and ascertain its safety.

## 1. Introduction

Rice (*Oryza sativa*) is the most important crop worldwide in terms of total production and the number of consumers who depend on it as a staple food [1]. It has been shown that rice and other cereals are susceptible to contamination by mycotoxin-producing fungi during the various stages of cultivation and processing [2–4]. These mycotoxins are produced by fungi, principally *Aspergillus, Penicillium, Fusarium,* and *Alternaria* [5], with over 300–400 different mycotoxins identified in a wide range of foods, including cereals, nuts, dried fruits, coffee, cacao, spices, legumes, and some fruits. However, they can also enter the food chain via bioaccumulation in eggs, milk, and the flesh of animals that eat contaminated foods. Accumulated toxins have also been found in other processed foods (bread, wine, beer, etc.) due to the use of contaminated ingredients [3, 5–7]. Some frequently reported mycotoxins include aflatoxins, ochratoxins, fumonisins, and trichothecenes. Others, such as patulin and citrinin, are also important, with recent studies suggesting that certain emerging mycotoxins such as fusaproliferin, beauvericin (BEA), enniantins, and moniliformin [8], whose proliferation is related to environmental factors including climate change, can also be considered relevant [9, 10]. A wide variety of analytical methods have been used to determine mycotoxins in foods, including immune-enzymatic tests (ELISA), fine-layer chromatography, capillary electrophoresis, and gas chromatography. However, the most popular technique is high-performance liquid chromatography (HPLC) with detection by UV/Vis, fluorescence (FLD), or mass spectrometry (MS) [11].

The use of vertical silos for cereal storage, sometimes equipped with temperature control and aeration, is a common practice for preserving grain quality and safety. However, even with the strictest control practices, stored cereals can present mycotoxin contamination [12]. In rice, there have been reports of the so-called field mycotoxins such as those produced by *Fusarium* due to accumulation at significant levels before drying and storage [13]. The so-called storage mycotoxins have also been reported, including those produced by the genera *Aspergillus* and *Penicillium,* which require lower humidity to boost growth and mycotoxin production [14].

One of the mycotoxins produced by *Fusarium* is zearalenone (ZEA), whose toxicity is associated with reproductive problems in some animals (and possibly in humans). This is due to its interaction with estrogen receptors, as it competes strongly with 17*β*-estradiol for binding to the cytosolic estrogen receptors present in the uterus, hypothalamus, and mammary and pituitary glands [15]. Some of the most studied storage mycotoxins are aflatoxins (AFB1, AFB2, AFG1, AFG2). AFB1 is the best known of these due to its mutagenic and carcinogenic properties in both humans and animals. It is known to cause hepatocellular carcinoma, growth suppression, immune system modulation, and malnutrition [16]. High AFB1 rates recently found in various countries' food supplies, especially in Africa and Asia, indicate that populational exposure to this toxin largely still has no adequate control [17]. Ochratoxin A (OTA) has been shown to be nephrotoxic, hepatotoxic, teratogenic, and immunotoxic for various animal species [18, 19]. One of the most important aspects of studies about mycotoxin presence is determining the degree of human exposure related to ingesting contaminated foods, especially from those considered to have high consumption levels [20]. The objective of the present study was to evaluate the potential presence of aflatoxins (AFB1, AFB2, AFG1, AFG2), ochratoxin A (OTA), and zearalenone (ZEA) in polished rice and paddy rice stored in silos located in Chiriquí, Panama. An evaluation was done on the food exposure of the adult Panamanian population, considering their rice consumption and determined levels of selected mycotoxins. As far as we know, this is the first report about determining these 6 mycotoxins in grains of rice using an HPLC-FLD methodology in Panama.

## 2. Materials and Methods

### 2.1. Chemicals and Reactives

Methanol, acetonitrile, acetic acid, and HPLC-grade water used for the mobile phases were provided by Merck (Germany). Individual patterned solutions for aflatoxins B1, B2, G1, G2, and zearalenone of 25 *μ*g/mL each, as well as ochratoxin A of 10 *μ*g/mL were bought from Trilogy (USA). Phosphate-buffered saline (PBS) was bought from Research Products International (USA). For sample extraction and cleaning, the experiment used Afla-OtaCLEAN immunoaffinity columns from LCTech (Germany) for aflatoxins and ochratoxin A, and Easy-Extract from R-Biopharm (Germany) for zearalenone.

### 2.2. Standard Solution Preparation

Based on the individual standard solutions, intermediate solutions were prepared for each mycotoxin with a concentration of 1.0 *μ*g/mL for aflatoxins in acetonitrile and 0.5 *μ*g/mL of OTA in methanol. These were used as the basis to prepare a mix of aflatoxins with a concentration of 200 ng/mL of AFB1 and AFG1, and of 100 ng/mL of AFB2 and AFG2, which was used along with the OTA solution (500 ng/mL) for preparing the standard solutions. The calibration solutions were prepared in a mixture of acetic acid 0.1% and methanol (50 : 50), with six levels of concentration in a range of 1.0 to 40.0 ng/mL for aflatoxins B1 and G1, from 0.5 to 20.0 ng/mL for aflatoxins B2 and G2, and 1.0 to 80.0 ng/mL for ochratoxin A. For ZEA solutions, based on the certified solution of 25 *μ*g/mL an intermediate solution was prepared at 1.0 *μ*g/mL. From this intermediate solution, the calibration solutions were prepared in a mixture of acetonitrile and water (50 : 50 v/v) with a concentration range of 12.5 to 600.0 ng/mL.

### 2.3. Sampling

A total of 23 samples (11 of paddy rice and 12 of polished rice) were gathered from the silos of 8 mills located in Chiriquí, Panama. Multiple sampling sites were selected and distributed in the conveyer belt located at the lower exit of the silo (paddy rice) and the packing line (polished rice). From each point, portions of around 100 g were extracted until they reached 4.0 kg. These were homogenized, and roughly 1.0 Kg was taken to grind and sift with a #20 mesh, to then be stored at −20°C until the analyses.

### 2.4. Mycotoxin Determination

#### 2.4.1. Aflatoxins and Ochratoxin A

**Figure 1:**
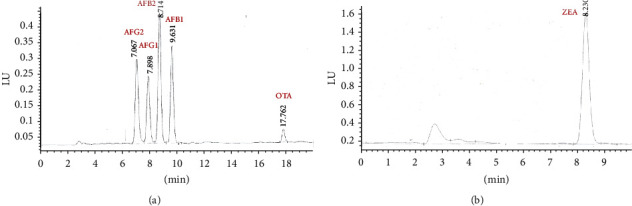
Characteristic chromatogram obtained for the separation of (a) AFG2, AFG1, AFB2, AFB1, and OTA in a standard mix of concentrations of 5.0 *µ*g/Kg for AFG1 and AFB1, 2.5 *µ*g/Kg for AFG2 and AFB2, and 5.0 *µ*g/Kg for OTA (b) standard solution of ZEA at a concentration of 200.0 *µ*g/Kg.

Following immunoaffinity column manufacturer instructions, 20 g of the sample was weighed and placed in a mixing cup at high velocity for five minutes with 100 mL of methanol and water (80 : 20). Next, the extract obtained was passed through a #4 Whatman paper filter. 14 mL were taken, to which 86 mL of PBS buffer solution was added (pH 7.2). 50 mL of this diluted extract was taken and passed through an immunoaffinity column, maintaining a maximum flow of 2 mL/min. Next, the column was washed, passing 10 mL of distilled water through it. Finally, 2 elutions were performed with 1 mL of methanol, letting the first addition of methanol act on the gel for 5 minutes. This extract was dried under a stream of nitrogen and reconstituted in 1.4 mL of a mixture composed of acetic acid 0.1%: methanol (50 : 50) for its injection into the HPLC. An Agilent 1260 Infinity chromatographic system was used (Agilent, USA), with a quaternary pump (G1311C), automatic injector (G7129A), thermostatized column compartment (G1316A), and fluorescence detector (G1321B). We applied post-column derivatization via an LCTech photochemical reactor (Germany). The separation process used a Zorbax SB-C18 reverse-phase column of 4.6 × 150 mm and 5 *μ*m (Agilent, USA), using the conditions proposed by Ainiza, Jinap, and Sanny [21] with modifications. The mobile phase consisted of 0.1% acetic acid (A), acetonitrile (B), and methanol. The gradient applied was 0 min = 60% A, 10% B, and 30% C; 14 min = 50% A and 50% B; and 20 min = 55% A, 30% B, and 15% C. The total run time was 20 min, with a post-run conditioning time of 10 min between each run. The injection volume was 100 *μ*L with a flow of 1.0 mL/min. The separation column temperature was programmed at 40°C. The wavelengths for the excitation wave (ex) and emission wave (em) were: ex = 364 nm, em = 455 nm (0 min); ex = 330 nm, em = 455 nm (15 min) [21].  Figure 1(a) shows the chromatographic separation of the analyzed mycotoxins.

#### 2.4.2. Zearalenone

According to the immunoaffinity column manufacturer's instructions, 25 gr of the sample were weighed and placed in a mixing cup at high velocity for two minutes with 125 mL of acetonitrile and water (75 : 25). Next, the extract obtained was passed through a #4 Whatman filter paper. 20 mL were taken from this, followed by adding 80 mL of PBS buffer solution (pH 7.4). 25 mL of this diluted extract was taken and passed through an easy-extract zearalenone immunoaffinity column (R-Biopharm, UK), maintaining a maximum flow of 2 mL/min. Next, the column was washed, passing 20 mL of PBS buffer solution through it with an approximate flow of 5 mL/min. Finally, an elution was done with 1.5 mL of acetonitrile and then with 1.5 mL of water to give a final volume of 3 mL. The HPLC system used was the type described in Section 2.4.1, without using derivatization. The mobile phase consisted of an isocratic flow of acetonitrile, water, and methanol (46 : 46 : 8). Total time for each run was 10 min, the injection volume was 100 *μ*L, and the flow was 1.0 mL/min. The separation column temperature was set at 40°C and wavelengths for excitation (ex) and emission (em) were 274 nm and 455 nm, respectively.  Figure 1(b) shows the chromatographic separation of ZEA.

### 2.5. Validation

The parameters evaluated for all the mycotoxins were linearity, sensitivity, exactness, and precision. Sensitivity was evaluated by the values for the limit of detection (LD) and limit of quantification (LQ). LD and LQ were calculated as a function of the signal/noise ratio (S/N) of 3 : 1 and 10 : 1, respectively, and evaluated for the curve of each mycotoxin analyzed. Linearity was evaluated by preparing the calibration curves with six concentration levels analyzed in triplicate. The recoveries and relative standard deviations (RSD) for the six mycotoxins studied were determined via fortifying blank samples from both matrices in three different concentrations and then analyzing them in 3 repetitions per day for 3 days. Method precision was determined via repeatability studies (*n* = 3) and reproducibility studies (*n* = 9) and expressed as the relative standard deviation (RSD %). Intradaily precision was expressed as the standard deviation of recovery values from the fortified samples measured on the same day (*n* = 3). Interdaily precision was determined by analyzing the enriched samples on three different days (*n* = 9). Complex matrices such as foods have components such as carbohydrates, proteins, or fats that significantly affect the determination of an analyte when a chromatographic method is used; this is known as the matrix effect. Therefore, the instrumental response may be enhanced or suppressed compared to solvent-based standards, resulting in an overestimation or underestimation of analyte concentration [22, 23]. The possible effect of the matrix on the analytical response was evaluated for the two matrices studied according to the procedure reported by Juan et al. [24].

### 2.6. Dietary Exposure Assessment

To perform a consumer risk evaluation for mycotoxin exposure, we used the zearalenone concentration levels found in the polished rice samples and the relevant consumption data, which were compared with the tolerable daily intake level (TDI) established by the General Directorate for Consumer Health and Protection of the European Commission, which is 0.25 *µ*g/Kg of body weight (bw) for ZEA [25]. Daily exposure dosage (DI) (*µ*g/Kg bw) was calculated according to the procedure reported by Reinholds et al. [26].

(1) $$\begin{array}{c} \text{DI}=\text{C}*\text{AC}*\frac{1}{\text{BW}}, \end{array}$$

where C refers to mycotoxin concentration (*μ*g/Kg) present in the rice; AC is the average consumption of rice (Kg) and BW is the estimated body weight (Kg) of the selected population group (70 kg for adults) taken according to EFSA recommendations.

### 2.7. Statistical Analysis

Statistical analysis was developed with ‘Rʼ (version 3.6.2) and ‘RStudioʼ (version 1.2.5033). Data normality was evaluated via the Kolmogorov–Smirnov test. The Kruskall–Wallis test was applied to determine whether there was any statistically significant difference between the datasets. The statistical significance was determined using a probability value of <0.05.

## 3. Results

### 3.1. Validation

The calibration curves prepared for each matrix presented determination coefficients (*R*^2^) greater than 0.9979 (Table 1). The LDs presented some variability between both sample types. The figures were between 0.10 and 0.25 *μ*g/Kg for aflatoxins and OTA, while for ZEA it was 9.35 *μ*g/Kg. The LQs varied between 0.25 *μ*g/Kg and 0.50 *μ*g/Kg for aflatoxins and OTA and 18.75 *μ*g/Kg for ZEA. Recovery from polished rice was between 78.4% and 103.2%, while for paddy rice, this was between 91.3% and 115.4%. Intraday precision evaluated as RSD% (*n* = 9) was below 20% in all cases.

**Figure 2:**
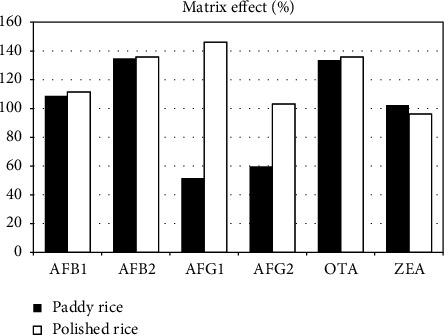
Matrix effect obtained for the six studied mycotoxins in polished rice and paddy rice.

The matrix effect was evaluated for the two types of samples (Figure 2). For polished rice, it ranged between 146.18% and 96.38%, and only the ZEA presented a slight suppression of the signal (96.38%); for paddy rice, the values were between 134.9% and 51.6%, and only AFG1 and AFG2 showed a signal suppression of 51.6% and 59.8% respectively.

### 3.2. Mycotoxin Occurrence

Of the samples analyzed in this study, 100% of the samples of paddy rice (11) were positive for at least one of the studied mycotoxins (Table 2). Zearalenone was found most often (90.91%) with a median of 440.14 *μ*g/Kg, followed by AFB1 (27.27%), AFG1 (18.18%), AFB2 (9.09%), and OTA (9.09%), while aflatoxin AFG2 was not found in any of the samples. Notably, zearalenone was found in concentrations of up to 1639.24 *μ*g/Kg.

For the polished rice samples (Table 2) the most common mycotoxin was zearalenone once again (50%), although in concentrations below the limits set in European legislation (100 *μ*g/Kg). Apart from zearalenone, the other mycotoxin found in these samples was AFB1, which was also in concentrations below the 2 *μ*g/Kg allowed by European regulation. OTA, whose maximum permitted value in rice for human consumption is 3 *μ*g/Kg, was not found in any of these samples [27].

### 3.3. Evaluation of Food Exposure

Food exposure was evaluated for ZEA since it was the most frequently found mycotoxin in the polished rice samples.  Table 3 shows the estimated values of daily ZEA intake in the adult Panamanian population from consuming contaminated rice. Daily intake (DI) values were found within a range of 0.03 to 0.07 *μ*g/Kg bw for the adult population of around 70 Kg. While these values are below the tolerable daily intake (0.25 *μ*g/Kg bw), it should be noted that other commonly consumed foods can also contribute to higher concentration levels, thereby increasing the total DI of ZEA.

## 4. Discussion

Due to cereals' importance and their susceptibility to the presence of mycotoxigenic fungi, the incidence of various mycotoxins in rice has been the subject of multiple studies which have used analytical techniques including ELISA and liquid chromatography with various detectors, as well as various cleaning and extraction methods including QuEChERS, SPE, immunoaffinity columns and more [7]. Zhao et al. analyzed 78 polished rice samples. 15.4% of these samples (12/78) were contaminated with AFB1. Of 22 samples of paddy rice, 4.5% (1/22) showed contamination with this mycotoxin [14]. Jettanajit and Nhujak analyzed 14 samples of brown rice, of which 21.4% (3/14) were contaminated with ZEA, while OTA did not appear in any of them [28]. Nazari et al. determined that 20 of these metabolites were present in 65 rice samples from Iran, and all the samples analyzed were contaminated with at least one mycotoxin [29]. Other studies which have also used chromatographic methods where mycotoxins were reported in rice have been done in Brazil (3/42, 6/44) [30, 31], Thailand (118/270) [32], Italy (180/180, 52/100) [33, 34], Morocco (6/21) [34], Slovenia (0/17) [35], Ivory Coast (76/88) [36], Pakistan (101/180) [37] and China (78/236, 4/10) [13, 38]. For Panama, our study shows the incidence (17/23) of grains of rice stored in silos located in the Chiriquí province, the region with the highest production in the country. Aflatoxins (B1, B2, G1, and G2), OTA, ZEA, DON, FB1, and FB2 have been the most studied mycotoxins in rice, mainly due to existent regulations. However, despite these substances' importance for public health, there are still many global regions where data about mycotoxin prevalence is limited or nonexistent, usually in developing countries or nations in crisis [39].

It should be noted that mycotoxins, especially ZEA, can occur in a variety of forms, known as “modified mycotoxins” that have the potential to pose an additional risk, as they can also have endocrine activity and contribute to the toxicity of the original mycotoxin alone, which poses additional challenges. More than 30 modified forms of ZEA have been described. However, these are not routinely tested [40]. These metabolites are formed by the fungus, the infested plant, and animals used for food production. Appropriate methods of analysis and sufficiently sensitive for their detection are mainly based on liquid chromatography (LC) with detection by mass spectroscopy (MS), since the use of conventional analysis methods (such as those used in this study) can be an underestimation of exposure by not considering these modified forms [41].

One noteworthy element is the prevalence of ZEA in both the samples of paddy rice (90.91%) and polished rice (50.0%), and the respective concentrations, which were up to 1639.24 *μ*g/Kg in paddy rice, while for polished rice the maximum ZEA value found was 24.37 *μ*g/Kg. This was the only one of the mycotoxins produced by *Fusarium* analyzed in this study. This contamination could have come principally from the field and not necessarily during storage, since these fungi generally grow and invade crops in fresh and wet field conditions during the blooming season [42]. Almeida et al. [43] reported high levels of ZEA concentration in byproducts (shell and powder) from the rice whitening and polishing process. Thus, high concentrations of this mycotoxin could be expected in rice samples with their shells, and following the polishing process, the zearalenone levels in the finished samples could be drastically lower. It should be noted that due to the thermostability of this mycotoxin, these low concentrations do not really imply elimination. Rather, the mycotoxin is distributed in the aforementioned byproducts. This makes it interesting to develop studies applied to rice byproducts or wastes since one of their most frequent applications is balanced animal feed. ZEA is also frequently involved in farm animal reproductive disorders, as well as hyperestrogenic syndromes in humans. There is evidence that ZEA and its metabolites have estrogenic activities in pigs, cows, and sheep [44].

Estimation of daily intake has provided evidence for adult population exposure to various mycotoxins due to consuming contaminated cereals and derivative products, representing a potential health risk [34]. Follow-up or monitoring data commonly follow individual chemical substances in raw food products and often do not provide a direct evaluation of dietary population exposure. There is little data in the literature about the behavior of prepared foods in analysis, which sometimes leads to an underestimation of the number of contaminants present in the food chain [45]. Although mycotoxin levels in rice and daily intake estimations have been evaluated in different studies, high rice consumption in the Panamanian population makes this an important contribution. Taghizadeh et al. estimated the daily intake of the Iranian population, with their review indicating that daily rice consumption in Iran is 110 g/day [46]. For the Panamanian population, daily rice consumption is 192 g/day [47]. While the DI obtained was below the TDI established by the General Consumer Health and Safety Directorate of the European Commission, this study did not estimate these data for different age groups due to the lack of available data about rice consumption among these groups in Panama.

## 5. Conclusions

This is the first report that has determined six mycotoxins in Panamanian rice via HPLC-FLD methodology. In the Panamanian population, rice consumption is high, and its susceptibility to the presence of mycotoxins makes it necessary to keep strict control over food safety when producing this cereal. Regulatory limit values vary by country, and current studies have shown a range of levels permitted for aflatoxins, ochratoxin A, and zearalenone in the analyzed rice samples. However, internal exposure may increase continuously over time due to high rice consumption, given these toxins' accumulation capacity in the human body. Mycotoxins can also be present in other food products in common daily diets, so mycotoxin levels can rise in the body due to other factors. Cooperative and continuous efforts are needed from government monitoring authorities and the scientific world to prevent and control the production of toxigenic fungi and assorted mycotoxins, as well as advance with available detection techniques in order to improve food safety.

## Figures and Tables

**Table 1:** Results for validation, sensitivity (limit of detection (LD) and quantification(LQ)), linearity (determination coefficients: *R*^2^, *x*: concentration, *y*: signal), and interday precision (*n* = 9, RSD: relative standard deviation) studied at three concentration levels.

Analyzed	Polished rice						
	LD (*μ*g/Kg)	LQ (*μ*g/Kg)	Equation	*R* ^2^	Rec ± RSD (%)		
					*n*1	*n*2	*n*3
AFB1	0.20	0.50	*y* = 0.8733*x* + 0.1618	0.9993	103.2 ± 14.2	97.9 ± 6.7	100.6 ± 3.1
AFB2	0.10	0.25	*y* = 2.7519*x* + 0.1752	0.9933	94.5 ± 13.3	97.1 ± 5.3	87.8 ± 1.2
AFG1	0.20	0.50	*y* = 0.8819*x* + 0.0706	0.9988	96.7 ± 2.6	101.2 ± 3.1	89.6 ± 4.4
AFG2	0.10	0.25	*y* = 1.3682*x* + 0.1805	0.9998	84.7 ± 13.3	89.7 ± 12.2	89.55 ± 9.1
OTA	0.25	0.50	*y* = 0.1761*x* + 0.1741	0.9991	78.4 ± 12.1	89.3 ± 9.8	85.9 ± 12.0
ZEA	9.35	18.75	*y* = 0.1238*x* − 0.1962	0.9997	97.06 ± 6.8	94.75 ± 1.7	95.61 ± 1.6

Paddy rice							
AFB1	0.20	0.50	*y* = 0.9529*x* − 0.0574	0.9998	104.1 ± 2.4	95.4 ± 1.0	93.88 ± 1.0
AFB2	0.10	0.25	*y* = 2.7299*x* − 0.2048	0.9998	115.4 ± 2.4	97.7 ± 0.5	91.3 ± 0.6
AFG1	0.20	0.50	*y* = 0.6033*x* + 0.1797	0.9979	102.3 ± 3.2	100.9 ± 1.5	110.3 ± 3.1
AFG2	0.10	0.25	*y* = 0.7925*x* + 0.4338	0.9982	111.9 ± 4.6	106.4 ± 5.6	94.4 ± 1.0
OTA	0.25	0.50	*y* = 0.1730*x* + 0.1526	0.9996	100.3 ± 13.4	96.92 ± 6.6	98.8 ± 2.0
ZEA	9.35	18.75	*y* = 0.1303*x* − 0.6474	0.9986	101.7 ± 4.4	103.3 ± 1.6	98.5 ± 3.3

**Table 2:** Incidence, range, and median of mycotoxins analyzed in rice samples, with shell and polished.

	Paddy rice					
	AFB1	AFB2	AFG1	AFG2	OTA	ZEA
Incidence (%), *n*	(27.27), 3	(9.09), 1	(18.18), 2	(0.00), 0	(9.09), 1	(90.91), 10
Range (*μ*g/Kg)	<LD-0.64	<LD-0.39	<LD-0.78	<LD	<LD-0.70	<LD-1639.24
Median (*μ*g/Kg ± DE)	0.29 ± 0.15	0.13 ± 0.08	0.25 ± 0.13	<LD	0.28 ± 0.11	440.14 ± 451.43

*Polished rice*						
Incidence (%), *n*	(25.00), 3	(0.00), 0	(0.00), 0	(0.00), 0	(0.00), 0	(50.00), 6
Range (*μ*g/Kg)	<LD-0.50	<LD	<LD	<LD	<LD	<LD-24.37
Median (*μ*g/Kg ± DE)	0.27 ± 0.11	<LD	<LD	<LD	<LD	12.06 ± 4.84
>OPL-EU (%)	0.00	0.00	0.00	0.00	0.00	0.00

>OPL-EU: over the permitted limit from the European Union.

**Table 3:** Estimation of daily zearalenone intake from eating contaminated rice.

Sample	ZEA concentration (*μ*g/Kg)	DI (adults) (*μ*g/Kg pc)	TDI (*μ*g/Kg pc)
9	14.94	0.04	0.25
11	10.89	0.03	
14	18.58	0.05	
15	24.20	0.07	
17	10.47	0.03	
18	11.49	0.03	

TDI: tolerable daily intake; DI: daily intake.

## Data Availability

All data are completely available for analysis and verification via the corresponding author at aravega@cwpanama.net.
